# Interrelationship between thyroid hormones and reduced renal function, a review article

**DOI:** 10.1186/s13044-024-00201-y

**Published:** 2024-07-15

**Authors:** Sadaf Agahi, Atieh Amouzegar, Mohammadjavad Honarvar, Fereidoun Azizi, Ladan Mehran

**Affiliations:** Endocrine Research Center, Research Institute for Endocrine Sciences, Shahid Beheshti University of Medical Sciences, P.O. Box: 19395-4763, Tehran, I. R. of Iran

**Keywords:** Free thyroxine, Free triiodothyronine, Thyroid stimulating hormone, Thyroid hormones, Chronic kidney disease, Glomerular filtration rate

## Abstract

**Background:**

Understanding the relationship of thyroid hormones with the development of chronic kidney disease (CKD) has important clinical implications for managing patients with both thyroid and kidney dysfunction. In this review, our purpose was to provide a thorough comprehension of the interplay between thyroid hormones, thyroid dysfunctions, and CKD.

**Summary:**

While there is evidence linking thyroid hormone levels to renal diseases, the association between thyroid hormones, specifically within the normal range, and the risk of CKD incidence is still a subject of debate. The Google Scholar, PubMed, Scopus, and Web of Science, were searched using the medical subject heading (MeSH) terms for the relevant keywords up to December 2023.

**Conclusion:**

Based on the review, the development of CKD is more consistently associated with higher serum TSH and thereafter lower serum free T3 levels; however, its association with free T4 is more controversial. Furthermore, subclinical and overt hypothyroidisms were considerably associated with incident CKD. Hyperthyroidism and Hashimoto thyroiditis might increase the risk of CKD.

## Introduction

In the 21st century, chronic kidney disease (CKD) is one of the emerging causes of death and illness worldwide [1] and is projected to be the 5th leading cause of years of life lost by 2040 [2]. CKD is associated with a high burden of comorbidities such as obesity, hypertension, diabetes, and cardiovascular disease [3]. While diabetes, hypertension, and certain genetic disorders are the most common causes of CKD, there is a significant proportion of cases where the underlying cause remains unknown [1].

Some research shows that thyroid hormones might affect kidney function, and kidney dysfunction could also contribute to thyroid disorders. Individuals who have any type of thyroid dysfunction are 1.64 times more likely to have reduced renal function compared to those who have normal thyroid function [4]. Hypothyroidism is more prevalent in CKD patients compared to the general population [5]. This is due to the direct and indirect effects of thyroid hormones on the kidney. Hypothyroidism negatively affects kidney function by disrupting the renin-angiotensin-aldosterone system (RAAS), reducing cardiac output, and causing vasoconstriction within the kidneys, ultimately leading to changes in renal blood flow. The effect of hyperthyroidism in CKD patients is not well established due to the limited number of research studies on this topic and the current results are controversial [6]. However, investigations regarding the association of thyroid hormones within normal ranges with CKD are limited. Thyroid dysfunction in CKD patients may be due to metabolic acidosis, malnutrition, chronic inflammation, and uremic toxins [5]. Moreover, the loss of thyroid hormones and thyroid-binding proteins in the urine may cause hypothyroidism [7]. In this narrative review, we searched the literature to find the complex interplay between thyroid hormones, thyroid dysfunctions, and chronic kidney diseases.

## Review

### **Triiodothyronine (T3) and CKD**

According to the cross-sectional study by Khatiwada et al., a significant positive correlation was found between free T3 and estimated glomerular filtration rate (eGFR) (*r* = 0.11, *p* = 0.036) [8]. This positive correlation between free T3 and eGFR was also validated by a Chinese study [9] and a German cohort study with 6 years of follow-up [10]. In the large cohort of German Chronic Kidney disease study on 4600 CKD patients conducted over four years, 1pmol/L increment in freeT3 levels was associated with a 2.99 mL/min/1.73 m^2^ increase in eGFR [11]. Similarly, a significant inverse association was observed between T3 and reduced kidney function in all participants despite their thyroid function in the Atherosclerosis Risk in Communities (ARIC) study (OR = 0.19, *P* < 0.001); however, after longitudinally analyzing with a median follow-up time of 19.6 years and adjustments for age, gender, nation, CRP, smoking, serum albumin, and body mass index (BMI), this association became insignificant [4]. In China, Li et al. studied 3563 subjects retrospectively and indicated that a 0.2 pg/ml increase in T3 level significantly reduced the risk of stage 1 to 4 CKD by 35–38%, while there was only a 2% risk reduction in stage 5 of CKD [12]. A South Korean prospective cohort of 104,633 euthyroid subjects followed for 3.5 years, controversially indicated that eGFR was decreased in higher levels of free T3 (*p* < 0.001); however, after adjustment for eGFR levels, increased levels of free T3 were negatively associated with the incidence of CKD with a hazard ratio of 0.95 and there was a significantly increased risk of CKD in serum free T3 levels below 3 pg/ml (*p* = 0.03) [13]. While most studies concluded higher free T3 levels are associated with increased eGFR and decreased CKD incidence, further large cohort studies with longer follow-up times are needed.

### Thyroxine (T4) and CKD

The results from cross-sectional studies investigating the association of T4 and CKD are controversial. Some studies found no association between free T4 and CKD [9, 11, 13–15]. While Juliane Peters et al. found a positive correlation between free T4 and eGFR [10], Schultheiss et al. showed a significantly inversed association between free T4 and eGFR [11]. Khatiwada et al. indicated a significant correlation between free T4 and eGFR (*r* = 0.119, *p* = 0.024) likewise free T3 [8]. According to the ARIC study, higher levels of free T4 were significantly associated with reduced kidney function, but prospectively, this association became much weaker after a median follow-up of 19.6 years [4]. X. Huang et al. studied 2103 subjects for four years and showed that higher levels of free T4 increased the risk of developing CKD by 88%; each 1-pmol/l increase in free T4 increased the risk of incident CKD by 12% and rapid eGFR decline by 10% [16]. A Chinese study with 3563 participants reported that a 0.3 pg/ml increase in free T4 level was associated with a 21% reduced risk of stage 5 CKD [12]. To make a summary, some studies showed a positive correlation between free T4 and eGFR and CKD incidence while some proved otherwise. Based on the evidence provided in each study none of them outweighs the other thus an absolute conclusion may not be possible.

### Thyroid-stimulating hormone (TSH) and CKD

Cross-sectional studies have found a negative association between TSH and eGFR [8, 14, 17]. In a study by Khatiwada et al., TSH was significantly and negatively associated with eGFR (*r* = − 2.01, *p* < 0.001) [8]. A study by Williams et al. on euthyroid patients indicated that TSH has a significant inverse association with renal plasma flow [18]. In the Rotterdom cohort study, higher TSH levels significantly decreased CKD incidence; however, no association was detected between TSH within the normal range and eGFR decline or incident CKD [14]. According to the ARIC study, TSH was positively associated with reduced kidney function but after a median follow-up of 19.6 years, the association disappeared [4]. In Japan, TSH showed a positive association with CKD with an odds ratio of 1.90 (p value < 0.001); longitudinally, the risk of CKD incidence in three years increased with higher levels of TSH (OR = 1.58, p value = 0.04) [19]. In a prospective cohort in South Korea, higher TSH levels were associated with an increased prevalence of CKD (HR = 1.59, *p* < 0.001) [13]. In a cohort of Japanese individuals with a 10-year follow-up, higher levels of serum TSH were significantly associated with increased risk of CKD in men (HR = 1.41, *p* = 0.02), but not in women [20]. Also, Li et al. indicated that there was an 8% increase in CKD stage 5 risk per 0.5 µIU/mL increment in TSH levels [12]. In a case-control study on diabetic patients, individuals with TSH levels higher than 3 µIU/mL had significantly higher CKD incidence in euthyroid patients (*p* = 0.027) [21]. In conclusion, all the studies showed higher TSH levels were associated with lower eGFR and increased CKD incidence among populations. Table 1 summarizes the impact of thyroid hormones on eGFR and CKD incidence in euthyroid subjects.

**Table 1:** Cross-sectional and cohort studies on the association between thyroid hormones and chronic kidney disease

First author(year)	Type of Study	Country	Sample size	Setting	Mean age	Follow-up Time	Result (TSH)	Result (FT4)	Result (FT3)	Result (TPO Ab)
Layal Chaker (2016)	cohort	Netherlands	5103	Population-based study (Rotterdam)	63.6	8.1 years	Negative association with eGFR and CKD incidence(OR = 0.85)	No association	Not assessed	Not assessed
X. Huang(2016)	cohort	Shanghai	2103	Community-based study	59.3	4 years	Not significant	Positive correlation with CKD incidence (OR = 1.88)	Not significant	Not assessed
Gordon Williams (2021)	cross-sectional	US	789	Population-based study	45.94		Negative correlation with RPF in euthyroids	Not assessed	Not assessed	Not assessed
Ulla T. Schultheiss (2017)	cohort	US	12,785	Community-based study (ARIC study)	57.4	19.6 years	No association	Positive association with incident CKD	No association	No association
Peggy Sekula (2020)	cohort	European	4600	Population-based study (German chronic kidney disease study)	60	4 years	Not assessed	No association	Negative association with renal events (ESRD, AKI, renal death)	Not assessed
Yasuji Arase (2019)	cohort	Japan	7609	Hospital-based study (Toranomon Hospital in Tokyo)	54	3 years	Higher normal TSH: positive association with incident CKD (OR = 0.86) Above normal TSH: positive association with incident CKD (OR = 1.58)	Not assessed	Not assessed	Not assessed
Eliseo Guallar (2014)	cohort	South Korean	104,633	Population-based study (Kangbuk Samsung Health Study)	38	3.5 years	Positive association with incident CKD ( baseline HR = 1.59, time-varying HR = 1.61)	No association	Negative association with incident CKD (HR = 0.95 )	Not assessed
Sijue Yang (2021)	case-control	China	2831	Hospital-based study (China Medical University)	51.08		Negative correlation with eGFR	Not assessed	Not assessed	Not assessed
Binbin Pan (2019)	cross-sectional	China	905	Hospital-based study	52.6		No association	No association	Positive correlation with eGFR	Not assessed
Keisuke Endo (2023)	cohort	Japan	10,392	population-based study	48	10 years	Positive association with incident CKD in TSH levels > 4.2 ( HR = 1.41) in men but not in woman	Not assessed	Not assessed	Not assessed

Thyroid hormones can affect kidney function through pre-renal changes which include cardiovascular regulations and shifts in renal blood flow (RBF), as well as direct renal changes such as GFR, tubular secretions and reabsorptions, and hormonal effects on tubular physiology. Additionally, thyroid hormones can regulate the expression of various transporters and channels in renal tubules, thereby influencing tubular excretion and reabsorption of water and electrolytes [22].

### Hypothyroidism and CKD

According to the ARIC study, Individuals with hypothyroidism had a 1.54-fold higher risk of reduced kidney function. However, this association was not significant [4]. Controversially, according to a Rotterdam study, individuals who have hypothyroidism are less likely to develop CKD compared to euthyroid individuals [12]. In a cross-sectional study in Japan which was performed at two stages in 1999 and 2017, the incidence of CKD in hypothyroid and euthyroid subjects above 55 years was assessed. It was shown that individuals with hypothyroidism were more prone to incident CKD (OR = 1.51, *p* < 0.001) compared to individuals with euthyroidism, and this association remained significant after adjustment for diabetes, hypertension, and demographics (OR = 1.25, *p* < 0.001) [23]. Furthermore, Chang et al. investigated the association of CKD with subclinical and overt hypothyroidism in a large population. They found that mean eGFR was 87.99, 83.46, and 72.22 mL/min/1.73 m^2^ in euthyroid, subclinical hypothyroid, and overt hypothyroid subjects, respectively (p-for-trend < 0.001); also, hypothyroidism and subclinical hypothyroidism (SCH) significantly increased the risk of incident CKD with respective OR of 3.16 and 1.74, even in fully adjusted models (*p* < 0.001) [24].

In 2017, a meta-analysis assessed the impact of SCH on CKD in diabetic patients. It indicated that SCH could significantly increase CKD incidence with an odds ratio of 1.80; also, SCH was significantly associated with CKD incidence in non-diabetic individuals (OR = 1.78, *p* < 0.001) [21]. Another meta-analysis showed that SCH increased the risk of CKD incidence with a pooled odds ratio of 1.37 (*p* = 0.000); however, in subjects older than 70 years it became insignificant [25]. In another nationwide cross-sectional study, individuals were evaluated for CKD and subclinical thyroid dysfunction. It indicated that SCH was significantly associated with CKD even after adjustments for gender, age, household income, education, smoking, alcohol use, activities including walking, abdominal obesity, high blood pressure, low HDL cholesterol, high triglyceride levels, high blood sugar, free T4, and thyroid peroxidase antibody (TPO Ab) (OR = 2.161, *p* = 0.041) [26].

In conclusion, based on large population-based studies mentioned above, hypothyroidism and subclinical hypothyroidism might be considered a risk factor for CKD. Thyroid medication could offer potential benefits for CKD patients [27].

Hypothyroidism has the potential to cause kidney dysfunction through both direct and indirect mechanisms. Hypothyroidism has a direct impact on the activity of the RAAS, which in turn affects the autoregulation of renal perfusion. Hypothyroidism can result in decreased cardiac output, decreased red cell production, increased peripheral vascular resistance, increased renal vasoconstriction, and declined renal vasodilators including vascular endothelial growth factor (VEGF) and insulin-like growth factor (IGF); these changes ultimately result in reduced GFR leading to CKD [4].

### Hyperthyroidism and CKD

The ARIC cohort study showed that hyperthyroidism also increased the risk of reduced kidney function by 1.40 times, but similar to hypothyroidism the association was not significant [4]. Also, Chaker et al. found that individuals with subclinical hyperthyroidism were more likely to have lower eGFR, while those with hypothyroidism had a lesser decline in eGFR [14]. A meta-analysis indicated that subclinical hyperthyroidism did not increase the risk of CKD incidence significantly (OR = 1.16, *P* = 0.115) [25]. As there are limited studies on this topic, further evaluations are essential to determine the exact impact of hyperthyroidism on CKD incidence. The summary of the effect of thyroid dysfunctions on CKD incidence and eGFR is provided in Table 2.

**Table 2:** Cross-sectional and cohort studies on the association between chronic kidney disease and thyroid dysfunctions

First author	Type of study	Country	publication year	Sample size	Setting	Mean age	Follow-up Time	Result (Hypothyroidism)	Result (Subclinical hypothyroidism)	Result (Hyperthyroidism)	Result (Subclinical hyperthyroidism)
Ulla T. Schultheiss	cohort	US	2017	12,785	Community-based study (ARIC study)	57.4	19.6 years	Not significant	Not significant	Not significant	Not assessed
Layal Chaker	cohort	Netherlands	2016	5103	Population-based study (Rotterdom)	63.6	8.1 years	Negative association with CKD	Not assessed	Positive association with CKD	Not assessed
Natsumi Matsuoka-Uchiyama	cross-sectional	Japan	2022	421	hospital-based study	61		Positive association with CKD incidence (OR = 1.51)	Not assessed	Not assessed	Not assessed
Yi-Cheng Chang	cross-sectional	Taiwan	2018	74,356	population-based			Positive association with CKD incidence (OR = 3.16)	Positive association with CKD incidence (OR = 1.74)	Not assessed	Not assessed
Xiaodong Wang	meta-analysis	China	2019	8 articles	population-based			Not assessed	Positive association with CKD incidence (OR = 1.37)	Not assessed	Not significant
Jian-Bo Zhou	case-control	China	2017	5530	hospital-based study	56.3	4 years	Not assessed	Positive association with CKD incidence (OR = 1.78)	Not assessed	Not assessed
Hye Jeong Kim	cross-sectional	Korea	2023	3257	population-based	43.34		Not assessed	Positive association with CKD incidence (OR = 2.16)	Not assessed	Not significant

One of the primary effects of hyperthyroidism on the cardiovascular system is increased cardiac output, heart rate, and consequent increased renal blood flow as a result. Glomerular hyperfiltration may lead to CKD progression [19]. Moreover, the increased sympathetic activity linked to hyperthyroidism can lead to the constriction of blood vessels in the kidneys, thereby exacerbating the impact on renal function [20]. Hyperthyroidism also increases sodium excretion in renal tubules thereby impairing water and electrolyte balance and impaired kidney function [21].

### Thyroid autoimmunity and CKD

The association of TPO Ab and CKD incidence is depicted in Table 1. The exact impact of thyroid autoimmunity on CKD is not clearly established as there are limited studies on this topic. A study on 1,401 Japanese women showed that the prevalence of CKD was significantly higher in women with Hashimoto thyroiditis (HT) than in those without HT. Also, patients with HT would experience a more severe form of CKD [22]. The association between TPO Ab levels and CKD is controversial. In a recent study on 5626 Iranian euthyroid individuals, the median level of TPO AB was significantly higher in CKD patients compared to the non-CKD group [18]. However, the association of TPO Ab and CKD was not significant in the ARIC study [9]. The chronic inflammation associated with Hashimoto’s thyroiditis may contribute to the development and progression of CKD by promoting oxidative stress and endothelial dysfunction. Moreover, chronic inflammation may damage glomerular and tubulointerstitial function, which are the main characteristics of CKD [22–24]. According to a study carried out in Italy, individuals who suffer from both Hashimoto’s thyroiditis and CKD have elevated levels of specific inflammatory markers, including C-reactive protein (CRP) and interleukin-6 (IL-6), when compared to those who only have CKD [23]. Further studies are needed to confirm these findings and to fully understand the underlying mechanisms.

### The effect of CKD on thyroid function

The effect of CKD on thyroid hormones has been summarized in Table 3. Hypothyroidism and subclinical hypothyroidism are the most common types of thyroid dysfunction observed in patients with CKD [6]. In Nepal, SCH was the leading type of thyroid dysfunction, which was found in 27.2% of patients in CKD patients. Overt hypothyroidism was the second most common type, affecting 8.1% of patients, followed by subclinical hyperthyroidism, which affected 3.3% of patients with chronic kidney disease [5]. Khatiwada et al. indicated that SCH was the most prevalent cause of thyroid dysfunction in CKD patients and it became significantly common with CKD progression [8]. In the Iranian population, the occurrence of SCH was found to be 7.3% in individuals with CKD, which was significantly higher than the prevalence of SCH in individuals without CKD, which was 5.2%. On the other hand, overt hypothyroidism was found to be 4.3% in CKD patients, which was significantly greater than the prevalence of overt hypothyroidism in those without CKD, which was only 1.6%. After adjusting for age, sex, smoking, TPO Ab positivity, and BMI, the likelihood of SCH was found to be higher in the CKD group compared to the non-CKD group. However, this difference did not exhibit statistically significance [18]. Rhee et al. found that there was a correlation between lower eGFR and an increased risk of hypothyroidism. Specifically, for every 10 mL/2 min/1.73 m decrease in eGFR, the risk of hypothyroidism increased by 18% and the TSH levels would be raised by about 0.11-mIU/L [28]. Narasaki et al. confirmed that T3 and free T3 were significantly lower in CKD stage 5 [6]. According to the Tehran Thyroid Study, Azizi et al. showed that T4 levels are lower in CKD patients, while TSH levels were higher in the Iranian population [29]. The cause of thyroid dysfunction in CKD patients is a complex issue that has not been fully understood. Decreased activity of deiodinase enzyme, lower inorganic iodide renal elimination, aberrant TSH response to thyrotropin-releasing hormone (TRH), uremic substance, metabolic acidosis, malnutrition, increased age, infected by HCV, chronic inflammation, and medications such as beta-blockers, amiodarone, and steroids could be potential explanations for impaired thyroid function in these patients [3].

**Table 3:** Cross-sectional and cohort studies on the serum thyroid hormones in patients with chronic kidney disease

First author(year)	Type of Study	Country	Sample size	Setting	Mean age	Follow-up Time	Result (TSH)	Result (FT4)	Result (FT3)	Result (TPO Ab)
Juliane Peters(2021)	cohort	Germany	4108	Hospital-based study	65.1	6 years	Not significant	Lower in CKD	Lower in CKD Increased low T3 syndrome in CKD	Not assessed
Fereidoun Azizi (2021)	case-control	Iranian	5626	Population-based study (Tehran Thyroid Study)	40.6		Higher in the CKD group	Lower in the CKD group	Not assessed	Higher in the CKD group
Saroj Khatiwada (2015)	cross-sectional	Nepal	360	Patients attending the biochemistry laboratory	44.1		Increased levels in stages 3–5 CKD	Not significant	Not significant	Not assessed
Jialin Li (2020)	case-control	China	3563	Hospital-based study	59		Lower in CKD patients in stages 1–2 (*p* < 0.05), and higher in CKD patients in stages 4–5 (*p* < 0.05).	Lower in CKD patients at all stages (*p* < 0.05)	Lower in CKD patients at all stages (*p* < 0.05)	Not assessed

Based on the physiology of T3 in CKD, the most amount of T3 is synthesized from the prohormone T4 in peripheral tissues. This conversion is catalyzed by type 1 and type 2 5’-deiodinase enzymes. Many factors related to CKD can contribute to a lower conversion of T4 to T3 in kidneys and other peripheral organs. One such factor is chronic metabolic acidosis, which affects the deiodination of iodothyronines, leading to a decrease in the peripheral conversion of T4 to T3. The expression of type 1 5’-deiodinase enzyme also is inhibited by the inflammatory cytokines like tumor necrosis factor (TNF)-α and interleukin (IL)-6, which is necessary for the conversion of T4 to T3 [4].

The majority of T4 in the bloodstream is attached to proteins, such as thyroid hormone-binding globulin (TBG), prealbumin, and albumin. Amon all, TBG is the most prevalent binding protein. It is notable that in various conditions in CKD, such as nephrotic syndrome and malnutrition, binding protein levels become low, and as a result, total T4 levels decrease. Although, further evaluations are required to fully comprehend the precise mechanisms by which free T4 functions in CKD [4].

Also, impaired clearance of TSH from the bloodstream, a longer half-life of TSH, reduced pulsatility of TSH secretion, altered glycosylation which affects the bioactivity of TSH, and a decreased response to TRH could explain the role of TSH in CKD patients [4].

Proteinuria also may result in significant losses of hormones and hormone-binding proteins. It has been indicated that CKD patients had higher levels of TSH, which was due to thyroid hormone loss in urine [30]. It was suggested that thyroid hormones and thyroid-binding proteins are wasted in the urine in CKD patients, leading to hypothyroidism [7].

## Summary

Overall the studies investigated in this review indicated that T3 might play a protective role in kidney function; whereas, higher TSH levels contributed to the development of CKD. The reports of the association of T4 and TPOAb with CKD are not conclusive. Subclinical and overt hypothyroidism is associated with the risk of CKD; however, hyperthyroidism has yet to be further investigated.

## Conclusion

Thyroid hormones could alter kidney function and reduced kidney function may be responsible for part of thyroid dysfunction. Thyroid hormone changes, even within normal range, might be associated with the development of CKD.

The management of patients with both thyroid and renal problems may be significantly affected by our growing understanding of the association between thyroid hormones and the development of chronic kidney disease. However, to determine the exact association of thyroid hormones with CKD, it is essential to conduct extensive population-based cohort studies with extended periods of follow-up time. In that case, a multidisciplinary approach is needed to improve patient outcomes and prevent the onset and progression of chronic kidney disease through screening and monitoring for early identification of thyroid dysfunction and individualized treatment.

## Data Availability

No datasets were generated or analysed during the current study.
